# What Has Bariatric Surgery Taught Us About the Role of the Upper Gastrointestinal Tract in the Regulation of Postprandial Glucose Metabolism?

**DOI:** 10.3389/fendo.2018.00324

**Published:** 2018-06-26

**Authors:** Jing Ma, Adrian Vella

**Affiliations:** ^1^Division of Endocrinology and Metabolism, Shanghai Renji Hospital, School of Medicine, Shanghai Jiaotong University, Shanghai, China; ^2^Division of Endocrinology, Diabetes and Metabolism, Mayo Clinic College of Medicine, Rochester, NY, United States

**Keywords:** incretin hormones, bariatric surgery, gastric emptying, gastric accommodation, insulin secretion, insulin action, vagus nerve

## Abstract

The interaction between the upper gastrointestinal tract and the endocrine system is important in the regulation of metabolism and of weight. The gastrointestinal tract has a heterogeneous cellular content and comprises a variety of cells that elaborate paracrine and endocrine mediators that collectively form the entero-endocrine system. The advent of therapy that utilizes these pathways as well as the association of bariatric surgery with diabetes remission has (re-)kindled interest in the role of the gastrointestinal tract in glucose homeostasis. In this review, we will use the changes wrought by bariatric surgery to provide insights into the various gut–pancreas interactions that maintain weight, regulate satiety, and limit glucose excursions after meal ingestion.

## Background

In the United States, the prevalence of obesity is rapidly increasing with 65% of adults and 17% of adolescents and children classified as being overweight or obese (1). Obesity is associated with multiple diseases, such as type 2 diabetes, non-alcoholic steatohepatitis, and osteoarthritis, as well as being associated with an increased frequency of the risk factors for cardiovascular disease (2). Approximately 9% of national health-care costs have been attributed to excess weight (3). Because of the evidence that weight reduction ameliorates or corrects the comorbidities of obesity, the US Preventive Services Task Force has recommended that body mass index (BMI) is routinely assessed and weight management recommended for obese patients (4).

Behavioral intervention with lifestyle and dietary modification usually achieves modest weight loss (4). While generally safe, most regain the weight lost within 5 years. Pharmacotherapy for obesity is considered for patients who have failed efforts at lifestyle modification and who have a BMI ≥ 30 kg/m^2^ or a BMI ≥ 27kg/m^2^ in the presence of comorbidities such as diabetes (5). However, there have been significant concerns about the long-term safety of such medications and many of the currently available medications have limited efficacy (6).

Bariatric surgery, sometimes referred to as metabolic surgery, is usually considered for patients who have a BMI ≥ 40 kg/m^2^ or a BMI ≥ 35 kg/m^2^ associated with comorbidities such as type 2 diabetes (5). Restrictive surgeries such as adjustable gastric banding (AGB) and sleeve gastrectomy (SG) limit the capacitance of the stomach. Roux-en-Y gastric bypass (RYGB) is the most commonly performed bypass procedure and produces gastric restriction together with selective malabsorption. RYGB involves creation of a gastric pouch by separating the stomach across the fundus. Drainage of this 10–30 ml pouch is achieved by a gastrojejunostomy. The distal end of the jejunum is anastomosed ~150 cm below the gastrojejunostomy effectively bypassing the distal stomach, duodenum, and proximal jejunum. Duodenal switch (DS) is a variation of biliopancreatic diversion and involves a SG with division of the duodenum below the pylorus. The distal ileum is anastomosed to the short stump of the duodenum producing a ~100 cm channel for nutrient absorption. The other end of the duodenum is closed and the remaining small bowel connected onto the enteral limb 75–100 cm from the ileocecal valve (2).

**Figure 1:**
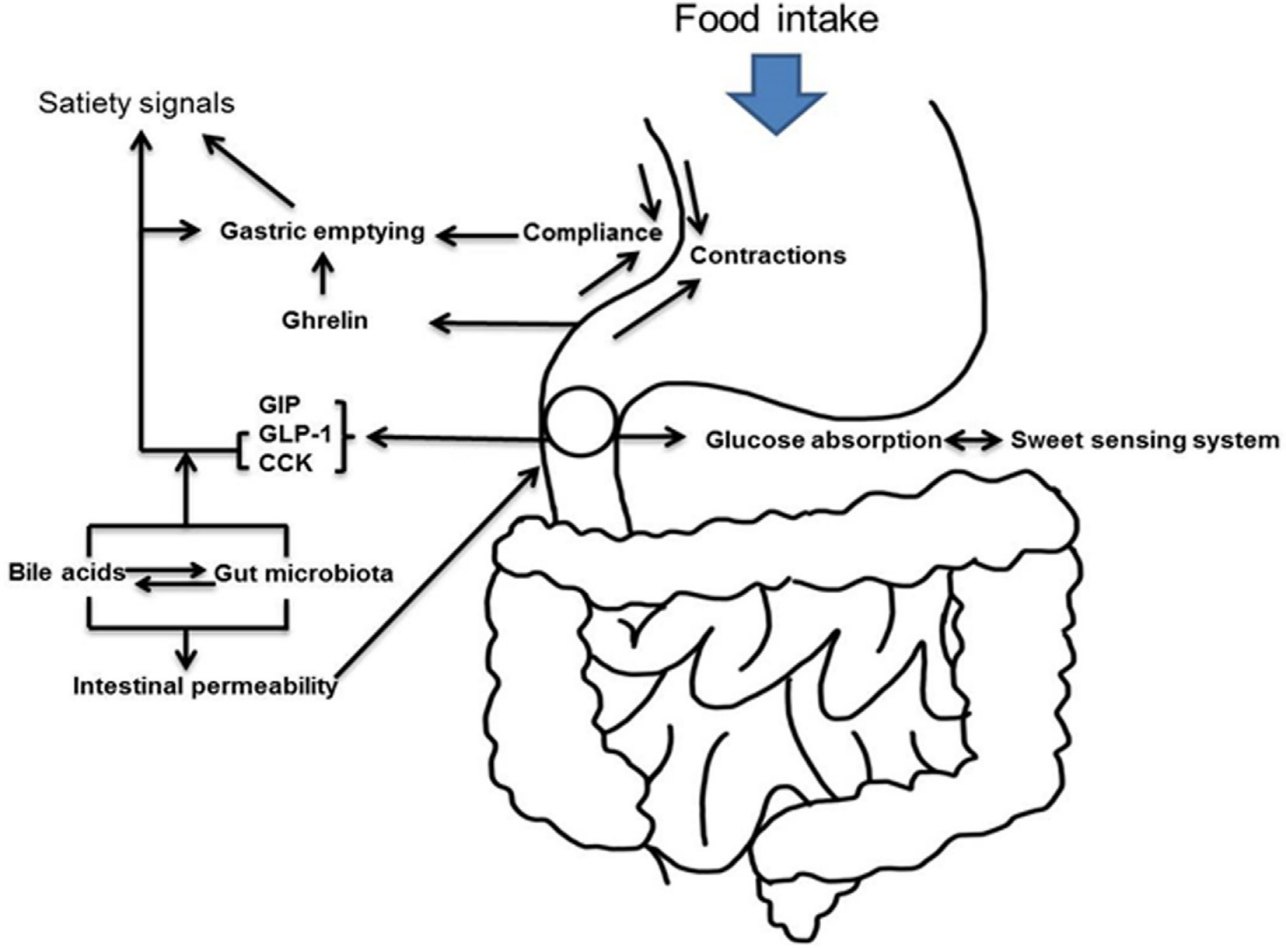
Schematic representation of upper gastrointestinal tract and hyperglycemia.

Observational and prospective studies have suggested that bariatric surgery is the most effective intervention for weight loss producing an average weight loss of 30–35% that is maintained in ~60% of patients at 5 years (7). This has led to a dramatic increase in the number of procedures performed annually from 13,365 in 1998 (8) to 216,000 in 2016 according to the data released by American Society for Metabolic and Bariatric Surgery (9). In a meta-analysis of 136 studies of bariatric surgery, which included a total of 22,094 patients, Buchwald et al. reported that within studies examining type 2 diabetes after bariatric surgery, 1,417 of 1,846 (76%) patients experienced complete resolution. When categorized by operative procedure, there were clear differences in efficacy. Diabetes resolved in 98.9% of patients undergoing biliopancreatic diversion or DS. In contrast, the rate was 83.7% for RYGB and 47.9% for AGB (10). A retrospective review of 257 patients who underwent the long-limb modification of RYGB (400–500 cm Roux limb length) at our institution reported resolution of type 2 diabetes in 94% of patients (11). Recent prospective, randomized controlled trials have, however, reported lower remission rates for diabetes with RYGB, although it remains superior to medical therapy (12–14). Setting aside the superiority of one procedure over the other in terms of inducing diabetes remission [which is likely related to residual β-cell function at the time of the procedure (15, 16) as well as the magnitude of weight loss (17)], obvious differences between procedures can be used to explore the role of the gastrointestinal tract in metabolism. RYGB is sometimes complicated by the occurrence of hyperinsulinemic hypoglycemia (18). Its incidence is uncertain although it has been suggested that excessive glucagon-like peptide-1 (GLP-1) secretion after RYGB (19) may be the cause of this phenomenon, but this is unlikely (20). The condition has been the subject of an extensive review recently (21) (Figure 1).

**Figure 2:**
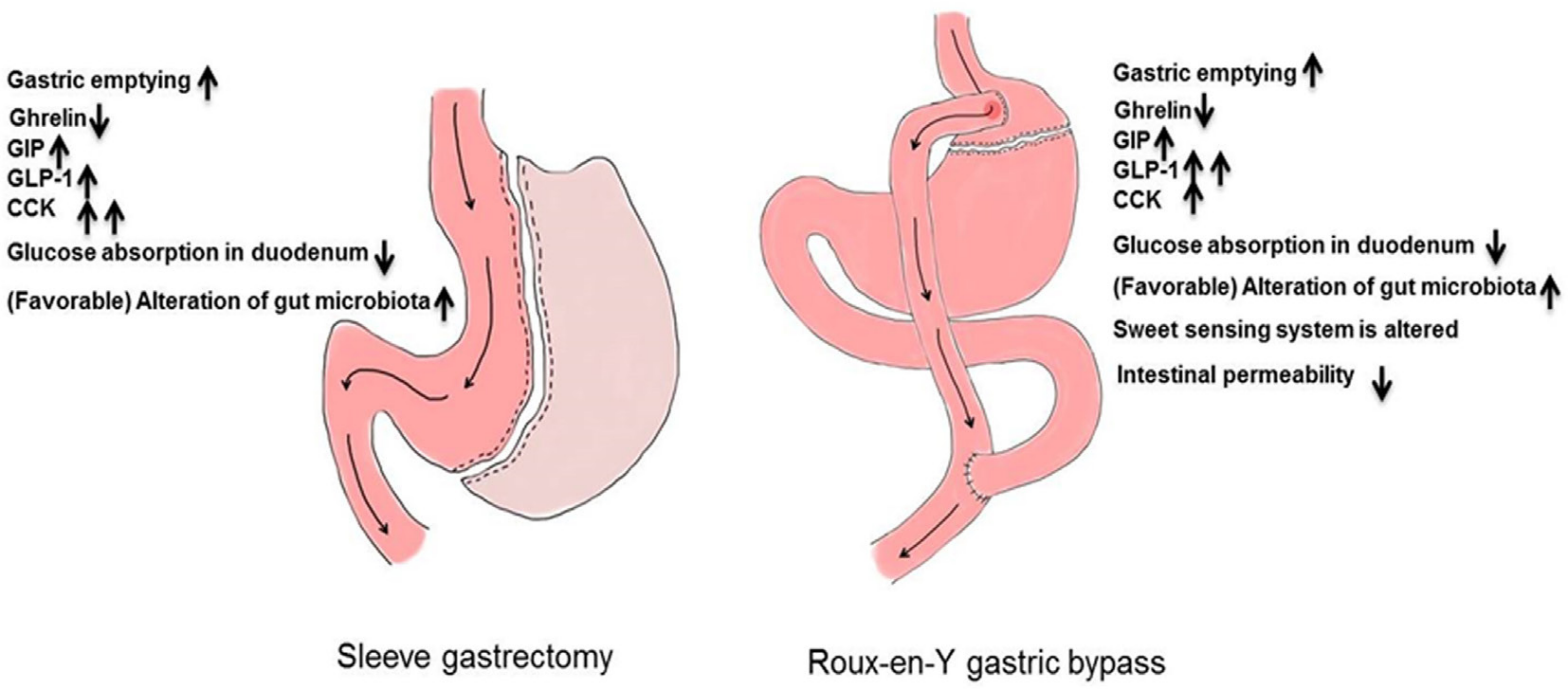
Upper intestinal adaptions after bariatric surgery.

The anatomic differences among bariatric procedures result in differences in enteroendocrine secretion (Table 1): postprandial GLP-1 concentrations are lower after SG compared to RYGB in the comparative studies undertaken in humans (22–26). On the other hand, a liquid meal, especially after gastric restriction, may not recreate conditions present after a solid meal (27). Indeed, liquid emptying especially after restrictive gastric surgery is dependent on fasting gastric volume (28, 29). SG removes the capacitance function of the stomach and decreases ghrelin concentrations to a greater extent than does RYGB (24, 25, 30). This is more apparent when acyl-ghrelin is measured (22, 31, 32). Whether these differences can explain metabolic outcomes will be explored in detail below (Figure 2).

**Table 1:** Efficacy of different bariatric surgeries.

Mechanism	SG	RYGB	AGB	DS
Weight loss	↓	↓	↓	↓
Amelioration of diabetes	71.6%	83.7%	47.9%	98.9%
Adverse effects	Band slippage, stoma obstruction, intractable postoperative vomiting	Dumping syndrome, dyspepsia, abdominal pain	Band erosion, leakage from the balloon	Gastrointestinal leaks and constipation
Plasma ghrelin	↓	↓	↓	↓
Plasma GLP-1	↑	↑	↑	↑
Plasma GIP	↑	↑	↔	N/A
Plasma CCK	↑	↑	↔	↑

*Increase, ↑; decrease, ↓; no change, ↔; NA, no available evidence; SG, sleeve gastrectomy; RYGB, Roux-en-Y gastric bypass; AGB, adjustable gastric banding; DS, duodenal switch; GLP-1, glucagon-like peptide-1; GIP, gastric inhibitory polypeptide; CCK, cholecystokinin*.

## Caloric Restriction—Changes in Gastric Volume, Compliance, and Accommodation

Restrictive procedures reduce gastric volume—indeed, the postoperative period of any form of bariatric surgery is characterized by a significant degree of caloric restriction (33). Fasting blood glucose and insulin resistance improves within 6 days of gastric bypass and occurs before any weight loss (34). This had been observed previously with very low-calorie diets outside of bariatric surgery (35), suggesting that caloric restriction at least partially, explains the acute improvement in glucose control after bariatric surgery (14, 36, 37). Six weeks of caloric restriction (700–900 kcal/day) decreases fasting and postprandial glycemia by lowering fasting endogenous glucose production and improving β-cell function (38). Jackness et al. showed that very low caloric restriction (500 kcal/day) produced a similar improvement in β-cell function as those who underwent post-RYGB (39). Indeed, caloric restriction after RYGB outweighs the effect of GLP-1 on glucose metabolism [as studied by the use of a competitive antagonist of GLP-1 at its receptor (40)]. Of course outside of the mechanical restrictions induced by bariatric surgery, there are difficulties with long-term compliance with regimens of caloric restriction. However, in an open-label, cluster-randomized trial 24% patients achieved weight losses of 15 kg or more in 12 months, and 86% experienced diabetes remission (41).

Multiple factors influence satiation including stomach capacitance and emptying. The stomach increases in volume in anticipation of food ingestion (42). In the normal stomach, this volume expansion is not associated with an increase in gastric wall stiffness (or increased intraluminal pressure) suggesting a change in the mechanical properties of the stomach wall (increased compliance) to accommodate the capacitive function of the stomach (43). This function is primarily fulfilled by the proximal stomach, which serves as a food reservoir, while the distal stomach triturates food to a size that can pass the pylorus. The physical nature, particle size, fat, and caloric content of food alter emptying rate (27). Although nutrient and non-caloric liquids empty rapidly, solids are initially retained in the stomach while antral contractions propel particles toward the closed pylorus. Food particles are emptied once they are ~2 mm in diameter (27). Restrictive procedures eliminate the function of the proximal stomach displacing food to the distal stomach and accelerating emptying. It is uncertain if restriction of accommodation alone alters gastric emptying (44). In SG, a functioning pylorus is retained and gastric volume is usually larger than the pouch created after RYGB. Post-RYGB it has been assumed that there is little neuromuscular control on gastric emptying since the pylorus is bypassed [although this may not be correct (45)]. Surgical vagotomy (which occurs in RYGB when the gastric pouch is created) alters gastric accommodation but may not change emptying (46) and typically does not have durable effects on weight (47).

Gastric emptying plays an important role in determining the magnitude of change in glucose concentrations after nutrient ingestion (48). Indeed, variation in the rate of gastric emptying alters peak insulin response after 75 g oral glucose, in both healthy subjects and patients with type 2 diabetes (49, 50). Fasting gastric volume affects the rate of emptying of a liquid challenge (51). Interventions that delay gastric emptying have the potential to regulate glycemia in patients with diabetes. Accelerating nutrient flow to the small intestine with erythromycin increases the postprandial glycemic response (52), whereas slowing gastric emptying with Xenin-25, a 25-amino acid neurotensin-related peptide, reduces postprandial blood glucose (53). Although it is important to match the rate of gastric emptying and the onset and offset of insulin action, significantly lower insulin requirements are observed in patients with type 1 diabetes with gastroparesis than those without, during the first hour of the postprandial period (54). Delaying gastric emptying is also a mechanism of action of some antidiabetic medications, such as GLP-1 analogs and pramlintide (55, 56).

Gut hormones can modulate food intake over and above that caused by mechanical restriction after bariatric surgery (22). Ghrelin increases food intake after esophagectomy or gastrectomy (57, 58). Neuronal GLP-1R mediates the anorectic effects of GLP-1 (59). Inhibition of GLP-1 action with Exendin-9,39 after RYGB accelerates gastric emptying (45). Taken together, these observations suggest that factors other than anatomy contribute to the upper gastrointestinal response to food ingestion. The attraction of certain foods decreases after RYGB (60) and appetite may be altered by enteroendocrine secretion (61, 62). A potential mechanism is *via* GLP-1, which alters gastrointestinal transit, gastric accommodation (45, 46, 63), and has direct effects on hypothalamic nuclei outside of the blood–brain barrier (64). GLP-1 and GLP-1 receptor agonists decrease food intake and cause weight loss (65, 66). GLP-1 also modulates taste sensitivity in rodents (67–70). The peripheral concentrations of GLP-1 observed in the early postprandial period in subjects post-RYGB, exceed concentrations observed after infusion at 0.75 pmol/kg/min, and are similar to those observed after infusion at 1.5 pmol/kg/min—both infusion rates that alter gastrointestinal function (71). It is, therefore, reasonable to consider that the postprandial rise in GLP-1 might affect feeding behavior after RYGB, and to a lesser extent SG, where the increase in GLP-1 is less marked (22–26). The elevated postprandial concentrations of GLP-1 observed after RYGB are unlikely to be the cause of diabetes remission after bariatric surgery. We (45) and others (72) have shown that inhibition of GLP-1 actions in the postprandial period has limited effects on glucose concentrations in people after RYGB. This is in agreement with data from mice deficient in the GLP-1 receptor that lost the same amount of weight as wild-type mice (73). This is also the case after SG in humans (74) and in mice deficient in the GLP-1 receptor (75). On the other hand, SG decreases acyl-ghrelin concentrations, presumably due to excision of a large part of the ghrelin-secreting stomach, which should decrease appetite (22, 31, 32). Fasting after SG is not associated with a rise in (low) ghrelin concentrations, in contrast to RYGB (22, 24, 25, 30–32).

In an effort to circumvent the costs and complications associated with bariatric surgery, various attempts have been made to develop endoscopically placed devices that might cause weight loss. One such device is a synthetic sleeve placed post-pylorus under endoscopic control. The rationale underlying such a device is to ensure that nutrients are prevented from coming in contact with the absorptive surfaces of the proximal small bowel (76). Unfortunately, such a device is prone to migration, bleeding, and bolus obstruction. A placebo-controlled study utilizing the device as treatment for type 2 diabetes was terminated prematurely because of a ~3% incidence of hepatic abscess in subjects using the device (77). Other devices such as intra-gastric balloons to induce early satiety are under study.

## Ghrelin

Ghrelin is a 28-amino acid peptide and is the only orexigenic hormone recognized in humans. It is secreted from the gastric mucosa and hypothalamus in both rodents and humans. There are two forms of circulating ghrelin, unacylated and acylated ghrelin (AG) (78). In the fasting state, AG is elevated (~110 pM) and decreases (~70 pM) in response to food ingestion. Patients with Prader–Willi syndrome—a syndrome characterized by excessive feeding behavior—have high concentrations of circulating ghrelin (79). Fasting and postprandial acyl-ghrelin levels are decreased following SG, compared to Roux-en-Y gastric bypass (RYGB), which may play a role in weight loss (26). SG involves removal of the gastric fundus—the primary source of ghrelin synthesis and secretion. Exogenous ghrelin administration increases energy intake in both rodents (80) and humans (81). Infusion of ghrelin in patients after esophagectomy (58) or gastrectomy (57) increases caloric intake and appetite. Although the contribution of ghrelin to normal physiology is unclear, it has been demonstrated that ghrelin can directly inhibit insulin secretion (82). Pharmacologic concentrations of ghrelin or ghrelin receptor agonists accelerate gastric emptying, suppress insulin secretion, and increase glucagon secretion (83). In a randomized controlled phase Ib clinical trial, ghrelin accelerated gastric emptying and improved gastrointestinal symptoms in patients with type 2 diabetes (84). Ghrelin receptor agonists are being developed as potential therapies for gastroparesis (85).

## Incretin Hormones

The incretin effect is a phenomenon first observed several decades ago when intravenous glucose produced lower insulin concentrations, despite higher glucose concentrations than observed after ingestion of an equivalent amount of glucose (86). This observation has subsequently been confirmed with isoglycemic infusion studies (87). The subsequent discovery of glucagon-like immunoreactivity in the gut led to the realization that proglucagon is synthesized in enteroendocrine cells intercalated between enterocytes and distributed throughout the intestine. GLP-1 and gastric inhibitory polypeptide (GIP) are two incretin hormones, which stimulate postprandial insulin secretion (88). GLP-1 is released from L-cells, most densely located in the distal small intestine and colon, although they are also located more proximally in the duodenum and jejunum (89). There is some evidence in rodent models of paracrine GLP-1 secretion within pancreatic islets (90). GIP is secreted from K cells (which reside mainly in the duodenum and upper jejunum) in response to nutrient ingestion. The early secretion of GLP-1 might involve an indirect neural or hormonal mechanism (91). The later secretion of GLP-1 is dependent on direct contact of nutrients in the small intestine with L-cells (92). Targeted delivery of lauric acid in enteric-coated pellets to the ileum and colon can stimulate substantial endogenous GLP-1 release and attenuate postprandial glycemia (93). To stimulate its receptor, GLP-1 requires the presence of 2 N-terminal amino acids, which are cleaved by the enzyme, dipeptidyl peptidase-4 (DPP-4), rendering the truncated form (GLP-1-9,36) inactive. Because of the widespread distribution of DPP-4, the active form of GLP-1 has a short half-life in the circulation (94). GLP-1 receptor agonists that are not substrates of DPP-4 and DPP-4 inhibitors are approved for the treatment of type 2 diabetes. They lower fasting and postprandial glucose concentrations (66). In addition to stimulating insulin secretion, pharmacologic concentrations of GLP-1 (and GLP-1 receptor agonists) inhibit gastric emptying, and suppress glucagon secretion. Moreover, GLP-1 and GLP-1 receptor agonists increase satiety, leading to a reduction in weight (95).

Although GIP secretion is preserved, the insulinotropic effect of GIP is diminished in type 2 diabetes. Unlike GLP-1, GIP stimulates glucagon secretion during hypoglycemia (96, 97) and has no effect on gastric emptying. Circulating concentrations of GIP are related to BMI (98), which suggests a role of GIP in energy metabolism. In mice, high GIP concentrations promote obesity and insulin resistance (99). However, recent study shows that there is a synergistic effect of GIP and GLP-1 co-agonists in weight lowering (100) and glycemic improvement in patients with type 2 diabetes than mono-agonist (101). Addition of a dual GIP/GLP-1 receptor agonist (NNC0090-2746) to metformin improved glycemic control with accompanying reductions in body weight and circulating cholesterol (102). The molecular mechanism underneath the metabolic improvements is not known. The effects of GIP on glucose metabolism are an area of ongoing investigation, which will hopefully be accelerated by the development of a specific GIP receptor antagonist (103).

## Cholecystokinin (CCK)

Cholecystokinin is secreted from the I-cells by exposure to nutrients in the duodenum and upper jejunum. Fat is a strong stimulus for CCK secretion, followed by protein, whereas carbohydrate is a weaker stimulus of CCK secretion. CCK concentrations increase from fivefold to tenfold after ingestion of a mixed meal and inhibit gastric emptying through activation of CCK-1 receptors (104). Physiological concentrations of CCK delay entry of glucose into the duodenum, reducing postprandial glucose excursions (105). In rats, CCK decreased hepatic glucose production to maintain glucose homeostasis by inhibiting CCK-A receptors and triggering a gut–brain–liver neuronal axis (106). In humans, CCK dose-dependently presents early satiety and reduces the energy intake at a buffet style meal, which was attenuated by the CCK-1 antagonist, loxiglumide (107). However, the long-term effects of CCK administration in humans and its role in obesity therapy are not clear.

## Role of the Vagus—Vagal Blockade/Vagotomy

The gastrointestinal tract is innervated by the parasympathetic and sympathetic divisions of the autonomic nervous system. The parasympathetic innervation originates from the dorsal motor nucleus of the vagus (DMV) in the medulla (108), while the sympathetic supply derives from the prevertebral ganglia (109). Gastric motility is partially controlled by the vagus nerve, a mixed motor, and sensory nerve. The sensory axons of the vagus receive afferent inputs from gastrointestinal receptors and then project to the nucleus of the solitary tract (110). Nucleus of the solitary tract (NTS) neurons activate vagal motor neurons in the nucleus ambiguus and the dorsomedial nucleus to regulate the smooth muscle contractions in the stomach and duodenum, with these neural loops being known as vagovagal reflexes (111). Bilateral truncal vagotomy (112), aiming for treating of peptic ulcer surgery, and electrical vagal blockade (113) results in delayed gastric emptying, and weight loss—at least in the short term. The gastric vagal branches are often damaged during bariatric surgery (114). It remains controversial whether vagal innervation of the portal hepatis contributes to the beneficial effects of RYGB on food intake, energy expenditure, and body weight (115). Electrical vagal blockade does not seem to have significant effects on glucose metabolism (116).

Obese subjects exhibit decreased heart rate variability likely due to an imbalance of sympathetic and parasympathetic activity (117). Overactivity of the sympathetic nervous system is more significant in obese subjects with type 2 diabetes than in those subjects without diabetes (118). Weight reduction following RYGB and AGB in severely obese patients is associated with an increase in heart rate variability (119). The underlying mechanism(s) remain unknown but the improvement in autonomic function does not appear to be related to improved insulin action (120). It has been posited that these changes in autonomic function could arise from crosstalk between the gastrointestinal tract and the central nervous system (121) generated by a neuro-inflammatory reflex (122) arising from the gut microflora.

## The Taste Signaling System

It is increasingly recognized that bariatric surgery may alter food preference and taste, in particular, the perception of sweet taste. This likely contributes to the reduction in energy intake after surgery (123). Both SG and RYGB result in a reduction of the frequency of food craving and the hedonic component of taste perception (124). Subjects experience a decreased desire to consume sweet and fatty flavors after RYGB (60) and SG (125).

The sweet taste signaling system includes heterodimeric G protein-coupled receptors, composed of the taste receptors (TRs), T1R2 + T1R3 heterodimers, which are activated by the binding of sweet compounds such as monosaccharides and disaccharides (126). These receptors are G-protein coupled (gustducin), and activation increases phospholipase C-β_2_ activity, which ultimately results in the release of Ca^2+^ from intracellular stores and the opening of a transient receptor potential ion channel TRPM5. The resulting membrane depolarization activates gustatory afferents (127). Sweet TRs are found in the tongue, gastrointestinal tract, pancreas, adipose tissue, brain, and bone (128). Expression of T1R2 + T1R3 also occurs in the entero-endocrine L cells (129), suggesting that the sweet sensing system in the gut is involved in incretin secretion. T1R3 knockout mice exhibit impaired GLP-1 secretion and glucose intolerance (130). Intragastric infusion of nutrients with lactisole, a T1R2/T1R3 blocker, attenuates GLP-1 and peptide YY secretion in humans (131, 132). The expression of sweet taste receptors and downstream molecule transcripts are disordered in models of type 2 diabetes (133). T1R2 expression is reciprocally regulated by luminal glucose in health, but not in patients with type 2 diabetes; during acute hyperglycemia, T1R2 transcript levels decrease in response to duodenal glucose infusion in healthy subjects, but increase in subjects with type 2 diabetes (134).

In addition to changes in oral taste sensitivity, the expression of T1R2 and T1R3 is decreased in the small intestine of rats after bariatric surgery; this occurs in parallel with elevation of GLP-1 (135). Functional magnetic resonance imaging or positron emission tomography demonstrates a decrease in neural activity in the brain reward areas in response to high-calorie foods (136).

## Permeability and Glucose Transport

The proximal small intestine initiates carbohydrate absorption after digestion. Glucose absorption is mediated by the sodium glucose co-transporter-1 (SGLT1) across the apical cell membrane and partially by the glucose transporter 2 (GLUT-2) at high glucose concentrations (137). The small intestine has a maximal capacity of glucose absorption of about 0.5 g/min (or 2 kcal/min) per 30 cm (138). The absorptive rate depends on the exposure rate of glucose, region, and length of the small intestine, and the expression of glucose transporters (139). The inhibition of motility and blood flow in the small intestine also attenuates glucose absorption (140). Plasma concentration of 3-O-methyl-glucose, a glucose analog that is not metabolized, is normally used to measure the absorption rate of glucose. Physiologically, enhanced glucose absorption in the proximal gut would increase blood glucose concentrations; acute hyperglycemia itself appears to enhance glucose absorption (141). Rodent models of diabetes exhibit small intestinal hyperplasia and increased absorption of glucose from intestinal mucosa (142). It is unclear to what extent inhibition of SGLT-1 can alter glucose absorption in a way that is relevant to postprandial glycemic control in diabetes.

Active glucose transport and intestinal permeability are increased in obesity and diabetes. For a given caloric intake, this could alter the nutrient load entering the portal circulation (143–146). Changes in intestinal thickness and transcription of SGLT-1 and GLUT-2 occur after RYGB (147, 148). Foregut exclusion decreases glucose absorption in rodents (149). However, it is currently not known, and if so, the extent to which RYGB and SG alter the rate of active intestinal glucose absorption or the rate of passive intestinal permeability.

Intestinal integrity provides a physical barrier to luminal bacteria, toxins, and antigens from the external environment. In health, it allows the passage of water and nutrients. Increased paracellular permeability, following disruption of the intestinal tight junctions enables bacteria to leak out of the intestinal lumen into the blood stream (150). Factors that influence permeability include the gut microbiome and fatty acids (whether ingested directly or as products of bacterial fermentation) (151). Bile acids could alter gut permeability through the G-protein-coupled bile acid receptor (TGR5), a cell surface receptor, which occurs at a high level expression in the human placenta and spleen and is also found in multiple tissues such as the lung, liver, adipocytes, and the gastrointestinal tract (152). A systematic review of 14 studies suggests that that fasting and postprandial lipopolysaccharide (LPS) are increased in patients with diabetes (153). LPS is the core component of the outer membrane of Gram-negative bacteria. Metabolic endotoxemia is defined by a twofold to threefold increase in plasma LPS concentration (154). Rosiglitazone is the most effective in the lowering the LPS in patients with type 2 diabetes, but the extent to which this contributes to the glucose-lowering effects of this compound are unknown (155).

## Bile Acid Metabolism

Bile acids are synthesized in hepatocytes *via* cytochrome P450-mediated oxidation of cholesterol and then secreted into the intestinal lumen through the biliary system. 95% of intestinal bile acids are reabsorbed in the distal gut and transported back to the liver by the enterohepatic circulation (156, 157). CCK induces production of bile, contraction of the gall bladder, and relaxation of the sphincter of Oddi, to deliver bile into the duodenum (158). Bile acids promote digestion and absorption of lipids in the gastrointestinal tract as well as participate in the regulation of glucose and energy homeostasis (159), acting through two specific receptors, the farnesoid X receptor (FXR) and TGR5.

FXR is expressed in the liver and the intestine in humans and is a member of the nuclear receptor super-family. It can be activated by both primary and secondary conjugated bile acids (160, 161). Similar to other nuclear receptors, FXR translocates to the cell nucleus and subsequently induces expression of the small heterodimer partner (SHP). SHP is involved in bile acids synthesis by downregulating the gene transcription of cholesterol 7 alpha-hydroxylase (CYP7A1), a rate-limiting enzyme in bile acid synthesis. The activation of TGR5 triggers the production of intracellular cAMP and secondary active the mitogen activated protein kinase signaling pathway to perform different functions in various organs. For instance, TGR5 is expressed in rodent and human pancreatic islets and regulates insulin secretion (162). TGR5 in enteroendocrine L cells stimulates secretion of GLP-1 (163). In addition, TGR5 may regulate energy homeostasis through activating deiodinases to convert the prohormone thyroxine (T4) into the active hormone triiodothyronine (T3) (164, 165).

Circulating bile acids’ concentrations after meal ingestion are decreased in obese subjects compared to lean controls (166). This difference is no longer significant after bariatric surgery (167, 168). The effects of SG on body weight and glucose tolerance are attenuated in the absence of FXR (169) and in TGR5 knock-out mice (170). In a diet-induced obesity mouse model, diversion of bile flow to the ileum produces similar metabolic benefits to RYGB (171), while the ability of RYGB to decrease body weight and improve glucose tolerance is substantially reduced in the absence of FXR. Bile acids may stimulate insulin secretion *via* activation of FXR and inhibition of ATP-dependent K + channels (172). It has been suggested that the changes in bile acid composition and concentrations induced by bariatric surgery can contribute to metabolic changes *via* FXR and TGR5-signaling pathways.

However, in humans, the contribution of bile acid changes to metabolic improvements after bariatric procedures is less clear. One study reported that total plasma bile acid concentrations increased twofold after RYGB but decreased after AGB, despite similar weight loss (173). Longitudinal study suggests that there are two phasic increases in plasma bile acid concentrations in a cohort of RYGB patients at 1 month and up to 24 months after surgery (168). This time course differs from the time course of metabolic resolution suggesting that they are unrelated phenomena.

## The Gut Microbiome

The human gut microbiome consists of 10–100 trillion of microorganisms, primarily bacteria, in the digestive tract (174). The composition of the gut microbiome influences digestion, absorption, inflammation, and intestinal motility. Over the past decade, several studies have demonstrated that gut microbial populations are closely associated with metabolic disorders such as dyslipidemia, obesity, and diabetes (175). The gut microbiome is established early in life (176). Exposure to antibiotics alters the normal distribution of intestinal flora and is associated with changes in metabolism in some (177) but not all studies (178). Diet and lifestyle and geography are the primary influencers of the distribution of intestinal flora (179).

In humans, gut microbiota produce glycoside hydrolases and polysaccharide lyases, which facilitate digestion of sucrose, lactose, and starch (180). Undigested polysaccharides are subject to fermentation by intestinal bacterial leading to the production of short-chain fatty acids, which can provide 5–10% energy consumption (181). Gut microbiota is also involved in signaling of FXR and TGR5 by modifying the bile acid pool (182). In fact, bile acids interact with gut microbiota by direct effects on the mucosal defense, membrane integrity, oxidative and pH stress to increase the growth of bile acid-metabolizing bacteria (183).

d-lactate acidosis is a rare complication of jejuno-ileal bypass surgery or patients with short bowel syndrome (SBS) (184). d-lactate production is mainly dependent on the colonic microbiome (184). Notably, in patients with SBS or after jejuno-ileal bypass surgery, delivery of an increased amount of undigested carbohydrates to the colon can result in excess d-lactate accumulation (185, 186). *Bacteroides thetaiotaomicron* abundance is decreased in obese subjects compared to lean individuals (187). Patients with type 2 diabetes may exhibit decreased abundance of butyrate-producing bacteria and an increase in various opportunistic pathogens (188). Dietary fiber intake in patients with type 2 diabetes increases acetate and butyrate-producing bacteria improves glycemic control (189). Use of metformin is accompanied by increased abundance of *Escherichia* and a decrease of *Intestinibacter* (190). Impaired glucose tolerance is reversed after the transfer of metformin-altered microbiota to germ-free mice (191). Acarbose alters bile acid metabolism through changes in gut microbial populations (192). This results in great interest in microbiota alteration on improvement of metabolic parameters. Recently, transplantation of fecal microbiota or “bacteriotherapy” seems a promising therapeutic method for metabolic syndrome (193).

Individuals with obesity exhibit markedly decreased abundance of *B. thetaiotaomicron*. However, the abundance of this microbe increased after SG despite similar metabolic outcomes suggesting that this is incidental to the improvements in glucose metabolism after bariatric surgery (187). Randomized trials are warranted in the future to further assess the gut mechanism after bariatric surgeries in humans.

## Conclusion

The upper gastrointestinal tract plays a primary role in the regulation of glucose excursions in response to meal ingestion by determining the rate of gastric emptying and indirectly by regulating appetite and satiation, barrier integrity, and nutrient absorption. Bariatric surgery has helped improve our knowledge of the mechanisms underlying gut–pancreas interactions and may enable development of effective dietary or pharmacological strategies in the management of diabetes.

## Author Contributions

JM wrote the paper with input from AV regarding content and layout. AV edited the draft for clarity and content.

## Conflict of Interest Statement

AV is an investigator in an investigator-initiated study sponsored by Novo Nordisk. He has consulted for vTv Therapeutics, XOMA, Sanofi-Aventis, Novartis, and Bayer in the past 5 years. JM has no relevant disclosures.
